# The power of weak ion-exchange resins assisted by amelogenin for natural remineralization of dental enamel: an in vitro study

**DOI:** 10.1007/s10266-022-00688-7

**Published:** 2022-02-11

**Authors:** Sandra Diez-García, María-Jesús Sánchez-Martín, Manuel Valiente

**Affiliations:** GTS Research Group, Department of Chemistry, Faculty of Science, Universitat Autònoma de Barcelona, 08193 Bellaterra, Spain

**Keywords:** Controlled release system, Amelogenin, Enamel remineralization, Dental caries, Fluorapatite

## Abstract

This study aims to develop an innovative dental product to remineralize dental enamel by a proper combination of ion-exchange resins as controlled release of mineral ions that form dental enamel, in the presence of amelogenin to guide the appropriate crystal growth. The novel product proposed consists of a combination of ion-exchange resins (weak acid and weak base) individually loaded with the remineralizing ions: Ca^2+^, PO_4_^3−^ and F^−^, also including Zn^2+^ in a minor amount as antibacterial, together with the protein amelogenin. Such cocktail provides onsite controlled release of the ions necessary for enamel remineralization due to the weak character of the resins and at the same time, a guiding tool for related crystal growth by the indicated protein. Amelogenin protein is involved in the structural development of natural enamel and takes a key role in controlling the crystal growth morphology and alignment at the enamel surface. Bovine teeth were treated by applying the resins and protein together with artificial saliva. Treated teeth were evaluated with nanoindentation, scanning electron microscopy and energy-dispersive X-ray spectroscopy. The innovative material induces the dental remineralization creating a fluorapatite layer with a hardness equivalent to sound enamel, with the appropriate alignment of corresponding nanocrystals, being the fluorapatite more acid resistant than the original mineral. Our results suggest that the new product shows potential for promoting long-term remineralization leading to the inhibition of caries and protection of dental structures.

## Introduction

Dental caries is a complex disease that afflicts a large proportion of the world’s population, regardless of gender, age and ethnicity, although it is more likely to be present in individuals with a low socioeconomic status [1–4]. Despite the fact that the prevalence of dental caries has descended over the last decades, the disease is still a major problem for adults and children, and an improved approach to prevention and therapy is currently needed [5]. Dental caries progresses when organic acids, produced by bacterial action from biofilms of dental plaque on dietary fermentable carbohydrates, diffuse into the tooth and dissolve the mineral [6–13]. Dental caries is a process, which can be stopped and reversed in its initial stages. If left untreated, the early reversible lesion can progress to the point of being irreversible [14, 15]. Another factor that puts oral health at risk and makes it necessary to find a solution is the popularity of the whitening systems and their side effects, such as dental erosion and hypersensitivity [16–19]. The mature enamel has no residual cellular components that can carry out the repair when it is damaged, so the restoration depends on physicochemical events at the tooth surface [1, 2]; here are protective factors that can prevent or reverse it, such as salivary proteins, calcium, phosphate, salivary flow and fluoride in saliva [5, 20, 21].

Enamel is the outer layer of the teeth and is the hardest and most mineralized tissue in vertebrates [22–25]. It is an acellular mineralized tissue comprised of densely packed crystallites of calcium hydroxyapatite organized into rod and interrod structures. Tooth enamel rods seem to extend from the dentino-enamel junction to the tooth surface and they are remarkably longer than wide. Interrods are interprismatic crystallites oriented in different directions [26].

During tooth germination, the enamel mineral is a carbonated apatite, not pure hydroxyapatite (HA). The mineral is related to hydroxyapatite but is much more soluble in acid, as well as calcium deficient (calcium is replaced by sodium, magnesium and zinc) and contains between 3 and 6% of carbonate ions replacing phosphate ions in the crystal lattice. Demineralization and remineralization is a dynamic process. The carbonate is preferentially lost during the demineralization process, and it is avoided during remineralization. Thus, the calcium-deficient and carbonate-rich regions of the crystal are markedly susceptible to be attacked during demineralization [5]. When the OH^−^ groups in pure hydroxyapatite (Ca_10_(PO_4_)_6_(OH)_2_) are completely replaced by fluoride ions (F^−^), it results in mineral fluorapatite (FA) (Ca_10_(PO_4_)_6_F_2_), which is really resistant to dissolution by acid. Mature enamel is mostly a mixture of hydroxyapatite and fluorapatite and for this reason, it is much less soluble than the original mineral [5, 21, 27].

Fluoride is widely used in dental products because primarily via topical mechanisms, it has a cariostatic effect inhibiting the demineralization at the crystal surfaces, enhancing the remineralization, interfering with plaque formation and inhibiting bacterial metabolism [5, 10, 21, 28–32]. The presence of fluoride ions restricts the formation of acidic, more soluble calcium phosphates as dicalcium phosphate dihydrate or octacalcium phosphate, and facilitates the creation of more acid-resistant fluorapatite or fluor-hydroxyapatite (partial substitution of fluoride by hydroxyl groups). Fluorapatite or fluorhydroxyapatite are formed when low levels of fluoride ions are present, and calcium fluoride will be created with high levels of fluoride ions. This calcium fluoride will hydrolyze to fluorhydroxyapatite in the presence of acid phosphate or phosphate ions. Moreover, fluoride is more effective in inhibiting hydroxyapatite dissolution when calcium and phosphate ions are also present in the solution [2]. With fluoride availability, demineralization is reduced because part of the calcium and phosphate lost by the dissolution of hydroxyapatite returns to the enamel as more acid-resistant fluorapatite [33].

Even though systemic fluoride by means of water fluoridation has been promoted in the past for the decline in dental caries, it has been found that the systemic benefits of fluoride are minimal and the primary reduction in dental caries is due to the topical effect of water fluoridation and the availability of fluoridated toothpastes. In several European countries without water fluoridation, a caries reduction has been seen after the introduction of fluoridated toothpastes [3]. Toothpastes are probably the most widespread products in health care and are one of the most effective ways to deliver free or soluble fluoride [34]. Nevertheless, high concentrations of fluoride can cause numerous side effects and due to the short oral application time of many fluoride products, fluoride ions are released rapidly producing high concentrations in a short time [21]. There has been an increase in the exposure to fluoride ingestion in children increasing the risk of toxicity and dental fluorosis due to the high levels of fluoride released into biological fluids [35].

A different approach to induce remineralization is used in the present study to avoid high concentrations of fluoride with its side effects and prolong the contact time between fluoride ions and teeth. The fluorapatite formation could be guided controlling the release speed of fluoride in the oral environment, in conjunction with the release of calcium and phosphate ions to induce the remineralization. For this purpose, a product (called NMTD [36], new material for dental treatment, from its Spanish acronym *Nuevo Material de Tratamiento Dental*) that provides a controlled release system for the anticaries treatment of dental tissues is used. NMTD is a combination of ion-exchange resins of weak acid and weak base ion-exchangers composition, loaded with calcium, fluoride, phosphate and zinc. The molar ratio of the ions has to be close to that of the organomineral tissue to be remineralized; in the case of the teeth, the approximate molar ratio between Ca^2+^, F^−^ and PO_4_^3−^, is 2:1:1, respectively. In contact with NMTD, the organomineral tissues are remineralized in an effective way, especially in the presence of Zn^2+^ ions. Zinc has two effects: it helps to combat the micro-organisms which cause caries due to its bactericidal properties against oral bacteria [37], and it is an initiator of the ionic release of the other structural ions. A toothpaste containing NMTD was proven to be effective in limiting in vitro enamel demineralization and in enhancing remineralization [38]. The application of ion-exchange materials has advantages compared to conventional chemical reagents, since the release of ions is only due to the ion-exchange mechanism and they do not introduce undesirable ions into the solution, they are characterized by practically neutral pH values and they can also adsorb bacteria on their surfaces. Most of these resins are non-toxic and are used in the pharmaceutical industry, in medical applications and also in the food industry [39].

On the other hand, the organic matrix composed of proteins secreted by the ameloblasts provides a scaffold for the enamel minerals to grow. But following this stage of development, the enamel enters a maturation phase in which most of the organic matter is degraded [23, 24, 40–42]. The protein amelogenin, that constitutes the 90% of the protein matrix, plays a central role in guiding the hierarchical organization of apatite crystals observed in mature enamel [22, 43–46]. It is known that the crystal morphology and alignment of enamel, as well as the correct enamel thickness, are the result of a protein-guided uniaxial growth process. The exact mechanism guiding this process remains undetermined due to the rapid degradation of the underlying protein matrix during tissue maturation. However, the role of self-assembly of enamel matrix proteins, particularly amelogenin, has been widely recognized as a key factor in controlling enamel structural development [22, 43, 47]. Therefore, there is considerable interest in the use of amelogenin and other similar self-assembling peptides for dental remineralization [48–54]. The aim of this study is to assess the NMTD remineralization capacity in the presence and absence of amelogenin to understand the influence of this protein on the crystal morphology and alignment during the remineralization process.

## Materials and methods

### Materials

Hydrochloric acid (37%) and potassium hydroxide pellets (85%) were purchased from Panreac (Barcelona, Spain). Chloramine T trihydrate (98–103%), HEPES (4-(2-hydroxyethyl)-1-piperazine-ethanesulfonic acid, 99.5%), magnesium chloride hexahydrate (99%), and calcium fluoride (95%) were purchased from Sigma-Aldrich (Steinheim, Germany); calcium chloride dihydrate (74–78%), potassium dihydrogen phosphate (99.5%), and tricalcium phosphate [35–40% (Ca)] from Panreac (Barcelona, Spain); potassium chloride (99–100.5%) from J. T. Baker (Deventer, Holland), all in powder form. Deionized water was purified through a Millipore purification system from Millipore (Milford, MA, USA). Reference sample of the hydroxyapatite was of analytical grade and was used as received without any further purification (> 90%, Fluka, Sigma-Aldrich, Steinheim, Germany).

The base of the weak acid ion-exchange resins is a copolymer from acrylic acid, divinylbenzene, and aliphatic diene with carboxylic acid functional groups (Lewatit S 8528, Lanxess, Leverkusen, Germany); and the base of the weak base ion-exchange resins is a styrene–divinylbenzene-copolymer with tertiary amine functional groups (Lewatit S 4528, Lanxess, Leverkusen, Germany). These food grade ion-exchange resins already charged with different ions (Zn^2+^, Ca^2+^, F^−^ and PO_4_^3−^) were purchased from MionTec (Leverkusen, Germany). They were ground to below 50 µm particle size and mixed to form the NMTD product, in which the corresponding molar ratio between Ca^2+^, F^−^ and PO_4_^3−^, is 2:1:1, respectively. Moreover, Zn^2+^ ions-charged resin is added, representing the 0.2% of the dry weight of the related resin.

### Methods

#### Reference fluorapatite sample

The fluorapatite reference powder was synthetized following a solid phase reaction [55] mixing in the agate miller calcium fluoride (95%) and tricalcium phosphate (35–40% (Ca)) at the ratio of 1.67 Ca/P. Subsequently, the reagents were placed in the muffle furnace (Selecta 366 PE, Barcelona, Spain) and heated to 1200 °C for 2 h. The solid FA was then grounded during 15 min into powder.

#### Mineral growth in solution

To form FA in solution, 0.6 g of NMTD product was mixed with 1 ml of artificial saliva (KCl 0.24 g/l, CaCl_2_∙2H_2_O 0.078 g/l, KH_2_PO_4_ 0.544 g/l, MgCl_2_∙6H_2_O 0.041 g/l, and HEPES 4.77 g/l; adjusted to pH 7.1 ± 0.4 with KOH pellets) and placed in a closed container inside an incubator at 37 °C during 24 h. The resultant paste is filtered with the aid of a funnel and a Kitasato flask under vacuum and dried at room temperature.

#### Amelogenin production

A human 175 amino acid amelogenin (Swissprot Q99217, isoform 1, excluding the signal peptide) was expressed in the *Escherichia coli* strain BL21 (DE3) and purified with an acid/heat treatment as described previously Svensson Bonde and Bulow [56]. The proteins were analyzed by matrix‐assisted laser desorption/ionization, sodium dodecyl sulfate polyacrylamide gel electrophoresis and western blot to confirm the obtainment of amelogenin. The recombinant amelogenin was quantified with a nanodrop.

Protein purification has been performed by the ICTS “NANBIOSIS”, more specifically by the Protein Production Platform of CIBER in Bioengineering, Biomaterials and Nanomedicine (CIBER-BBN)/IBB, at the UAB SePBioEs scientific-technical service (http://www.nanbiosis.es/portfolio/u1-protein-production-platform-ppp/).

#### Specimen preparation

Bovine mandibular incisors have a great macroscopic and microscopic similarity to human teeth, so they have been commonly used as a model in dental studies [57–59]. Human and bovine incisors have certain small differences that are important to consider when conducting physical or chemical studies of dental specimens. For example, bovine enamel crystals present larger diameter than human enamel crystals and bovine prisms differ in shape compared to human prisms. In addition, calcium distribution is more homogeneous in bovine enamel than in human enamel. However, there are no significant differences in fluoride uptake or in carbonate content between human and bovine enamel. Moreover, both kinds of teeth have also similar hardness, porosity and amount of interprismatic enamel [60–63].

Specimens of deciduous bovine incisors were cleaned of gross debris and the root was sectioned using a diamond saw (South Bay Technology, San Clemente, CA, USA). Samples were embedded in a Fastray acrylic resin (Harry J. Bosworth, Skokie, IL, USA) to close the root hole. Then, teeth were etched with 1 M HCl for 30 s [64] to simulate the early stage of dental erosion and cleaned by rinsing with MilliQ water while brushing for 20 s with an electric toothbrush Vitality (Braun Oral-B) with brush head EB20 (Braun Oral-B).

#### In vitro remineralizing treatment

Groups of six teeth were prepared as explained in “Specimen preparation” for each time and treatment: NMTD, NMTD with human amelogenin, and blank samples.

Blank samples were immersed in the artificial saliva solution (prepared as explained in “Mineral growth in solution”) at 37 °C during the different times of the experiment and cleaned with MilliQ water while brushing with the electric toothbrush for 20 s every 24 h.

**Fig. 1:**
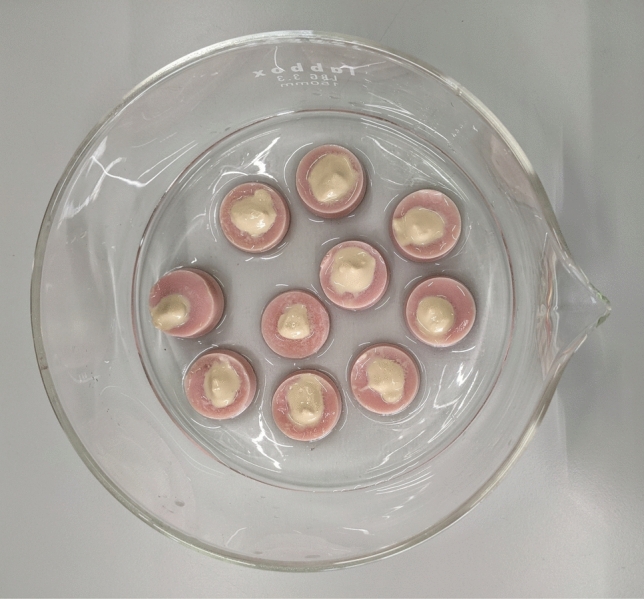
Bovine teeth with NMTD treatment placed in the container with artificial saliva on the bottom prior to sealing

For both remineralizing treatments, artificial saliva (in the presence or absence of 100 µg/ml of human amelogenin) was added to NMTD until obtaining the optimal consistence of thick gel for its application onto the tooth (about twice as much artificial saliva is added as NMTD resin). The mixture was applied directly on the enamel surface of the etched teeth (Fig. 1). The samples were placed in a closed container with artificial saliva on the bottom and inside an incubator to maintain the humidity and the natural temperature of the oral cavity (37 °C). Every 24 h, each treatment was renewed by carefully washing the specimens with MilliQ water and brushing with the electric toothbrush for 20 s before applying a fresh portion of treatment.

**Fig. 2:**
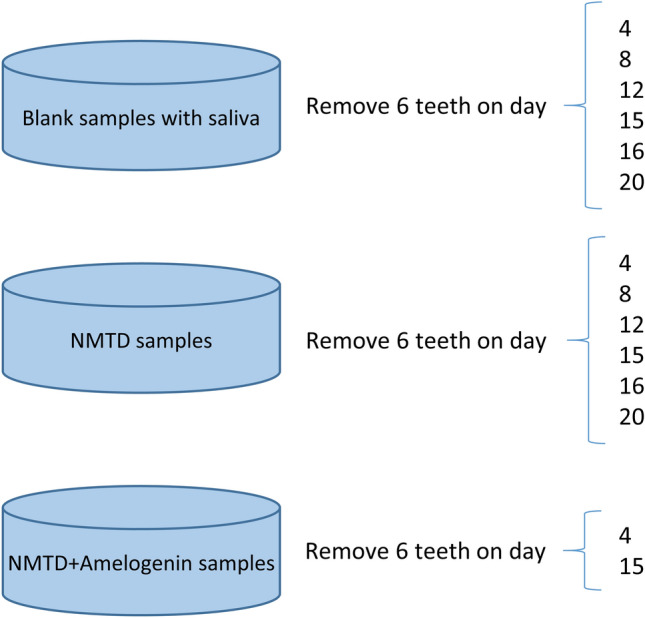
Scheme of the teeth with the different treatments indicating sample collection times

The different groups of samples were treated between 4 and 20 days (Fig. 2). Finally, teeth were cleaned by brushing with MilliQ water for 20 s using the electric toothbrush and stored in a 0.5% chloramine T solution until their analysis.

Moreover, some samples that were treated during 15 days with NMTD were brushed continuously for 15 min with the electric toothbrush Vitality (Braun Oral-B) with brush head EB20 (Braun Oral-B) maintaining constant force to study the strength of the remineralization.

To study the remineralized layer, selected samples were longitudinally cut in two halves along the central lobe with a Struers Minitom precision diamond saw (Copenhagen, Denmark) and polished with a Struers LaboPol-25 polisher (Copenhagen, Denmark). A sequence of silicon carbide paper was used to polish the longitudinal side, starting at grit size P1000 and sequentially increasing to P4000, under a constant flow of water. Then, a water-based diamond suspension containing monocrystalline diamonds and cooling lubricant with a mean particle size of 1 µm (DiaDuo-2, Struers, Copenhagen, Denmark) was used to finish the polishing. After each polishing, the samples were sonicated for 1 min to clean the polishing residues.

#### SEM–EDX measurements

The structure and elemental composition of the samples were analyzed by scanning electron microscopy (SEM) and energy-dispersive X-ray spectroscopy (EDX) with a Zeiss Merlin field emission SEM equipped with an EDX Oxford INCA X-Max detector. This microscope has a unique charge compensation system that allows the high-resolution imaging of non-conductive samples, and electrons which accumulate on the sample surface are swept away by a fine jet of nitrogen. The images were taken on the surface and the longitudinal section of the teeth with the secondary electron detector with a low voltage of 1–3 kV and the EDX measurements were taken at 10 kV. The powder from the mineral growth experiment and the HA and FA references were characterized under the same conditions as described above. All the experiments were performed at room temperature.

#### Nanoindentation

The hardness of the teeth was measured using an MTS Nano Indenter XP with a Berkovich tip that provides a fast and reliable way to acquire mechanical data on the submicron scale. The continuous stiffness measurements (CSM) were performed in the longitudinal section of the tooth with a depth limit of 1000 nm and a Poisson’s ratio of 0.25 [65, 66]. Continuous stiffness measurement with depth, in conjunction with the known indenter tip area function, allows continuous hardness monitoring [67]. All the experiments were performed at room temperature. Before every measurement, the Berkovich diamond indenter was calibrated on a standard fused silica specimen.

#### Software

MEDUSA program (Stockholm, Sweden) [68] was used to create the chemical equilibrium diagram of the species formed in the presence of the ions released from the NMTD. The basic parameters that are necessary for the calculation of distribution diagrams, including equilibrium constants, are included in the program database.

The SEM images treatment was done using ImageJ [69, 70], a public domain image processing and analysis program developed at the US National Institutes of Health.

GraphPad Prism 9 (GraphPad Software, San Diego, CA, USA) was used for statistical analysis.

## Results

### Mineral growth in solution

**Fig. 3:**
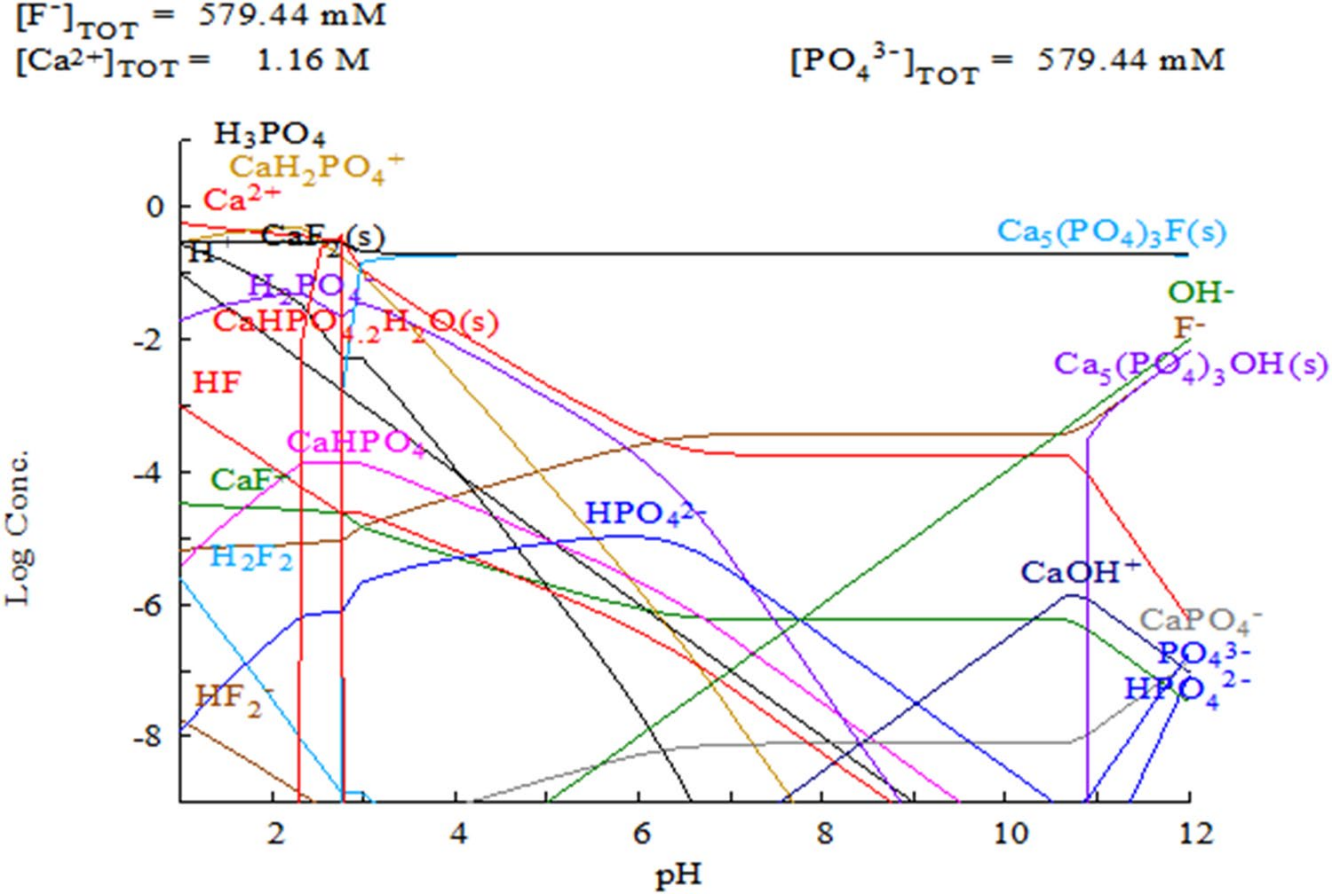
Theoretical distribution diagram of the species formed in the presence of the released ions from NMTD as function of medium pH (artificial saliva solution in our case)

Theoretically, the predominant species formed in the presence of the ions released from the NMTD at pH 7.1 would be calcium fluoride and fluorapatite, as it can be seen in the chemical species distribution diagram of Fig. 3.

**Fig. 4:**
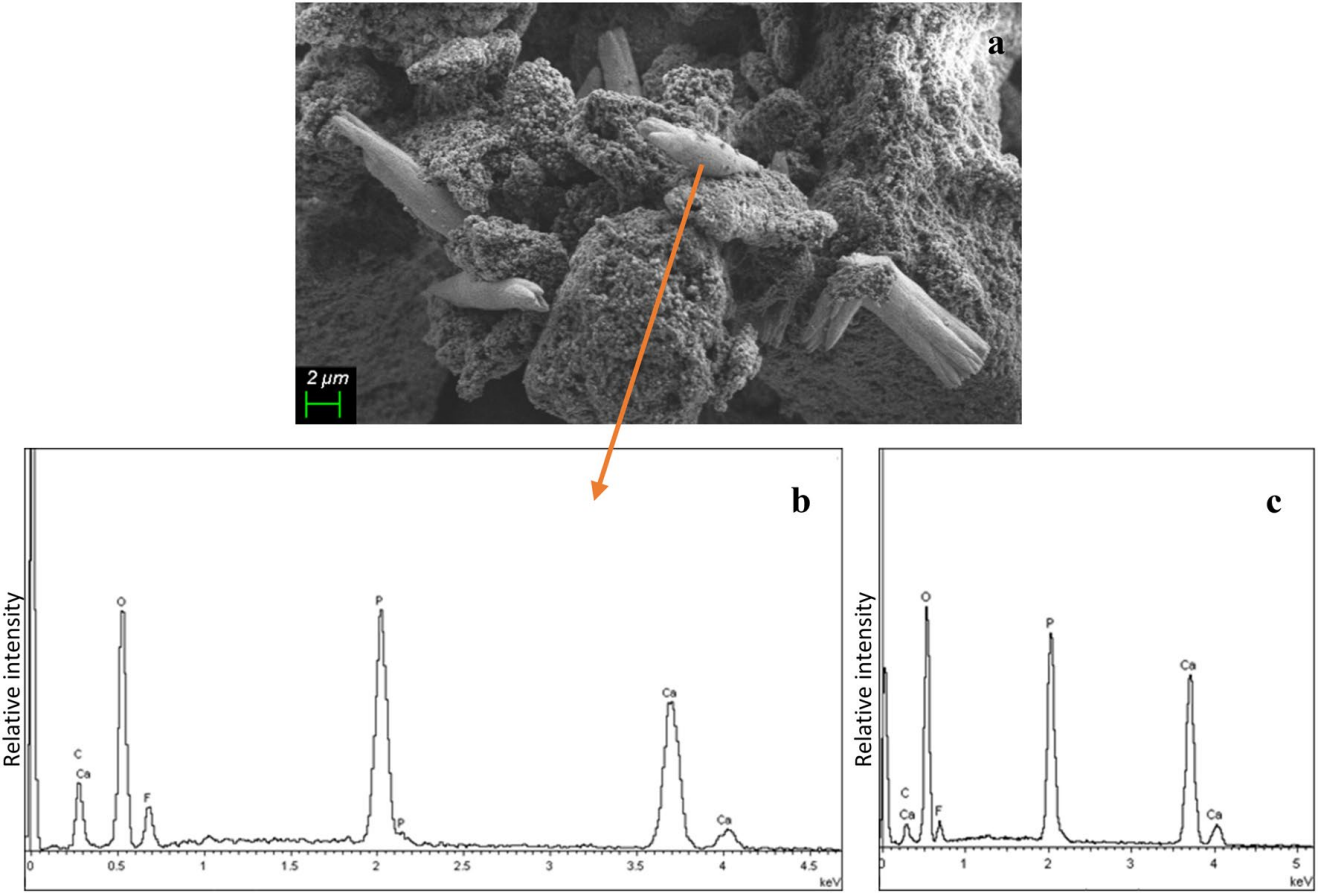
SEM image of the crystals obtained after the incubation of NMTD in artificial saliva solution during 24 h with 5000X magnification (**a**), EDX spectrum of one of the crystals (**b**), EDX spectrum of the fluorapatite reference powder (**c**)

Figure 4 shows the SEM image of the elongated crystals obtained after the incubation of NMTD in artificial saliva solution for 24 h and the EDX results of one crystal and the fluorapatite reference powder.

### In vitro experiment

#### SEM–EDX measurements

**Fig. 5:**
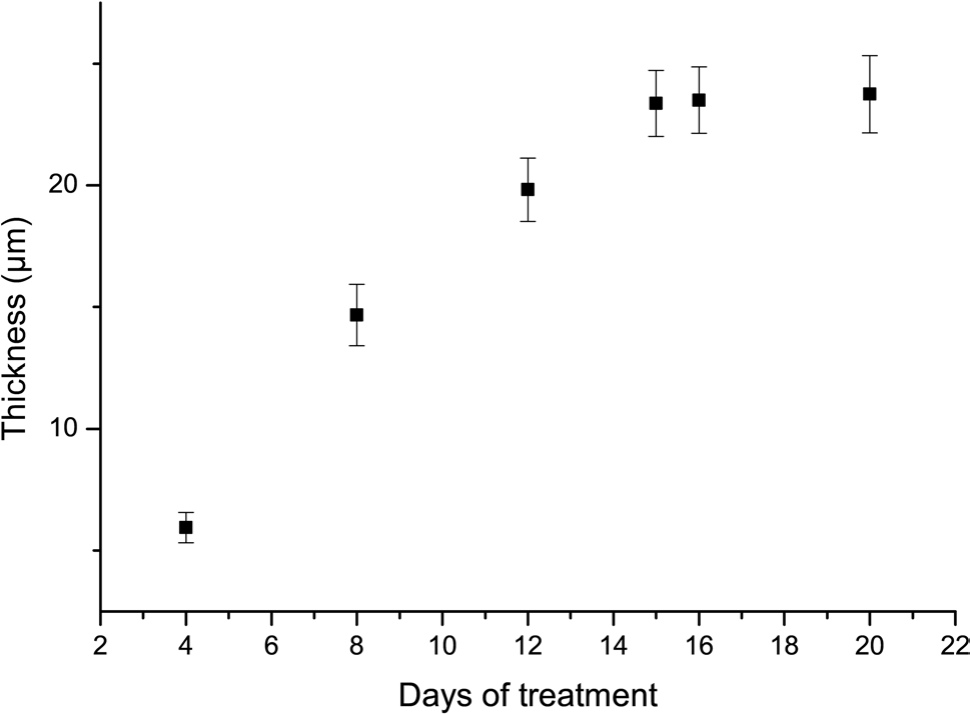
Evolution of the remineralized layer thickness with the time of NMTD treatment

Teeth were lengthwise cut and the remineralized layer thickness after each treatment time was measured using ImageJ software. Figure 5 shows that the layer grows gradually until day 15, when it reaches a plateau around 23 ± 1 µm.

**Fig. 6:**
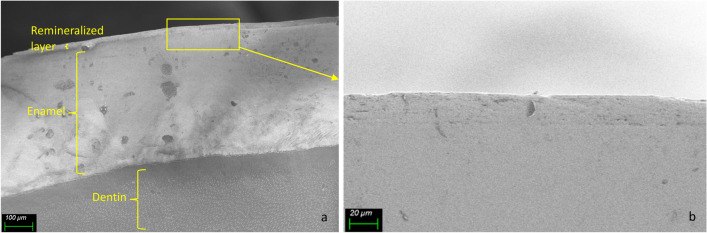
Longitudinal section SEM images of a sample treated with NMTD for 15 days and brushed for 15 min with × 250 magnification (**a**) and × 1000 magnification of the square area (**b**)

To study the formed layer bonding strength to the enamel surface, teeth were brushed continuously for 15 min after 15 days of treatment. Figure 6 represents a longitudinal section of a tooth showing the remineralized layer after brushing.

**Fig. 7:**
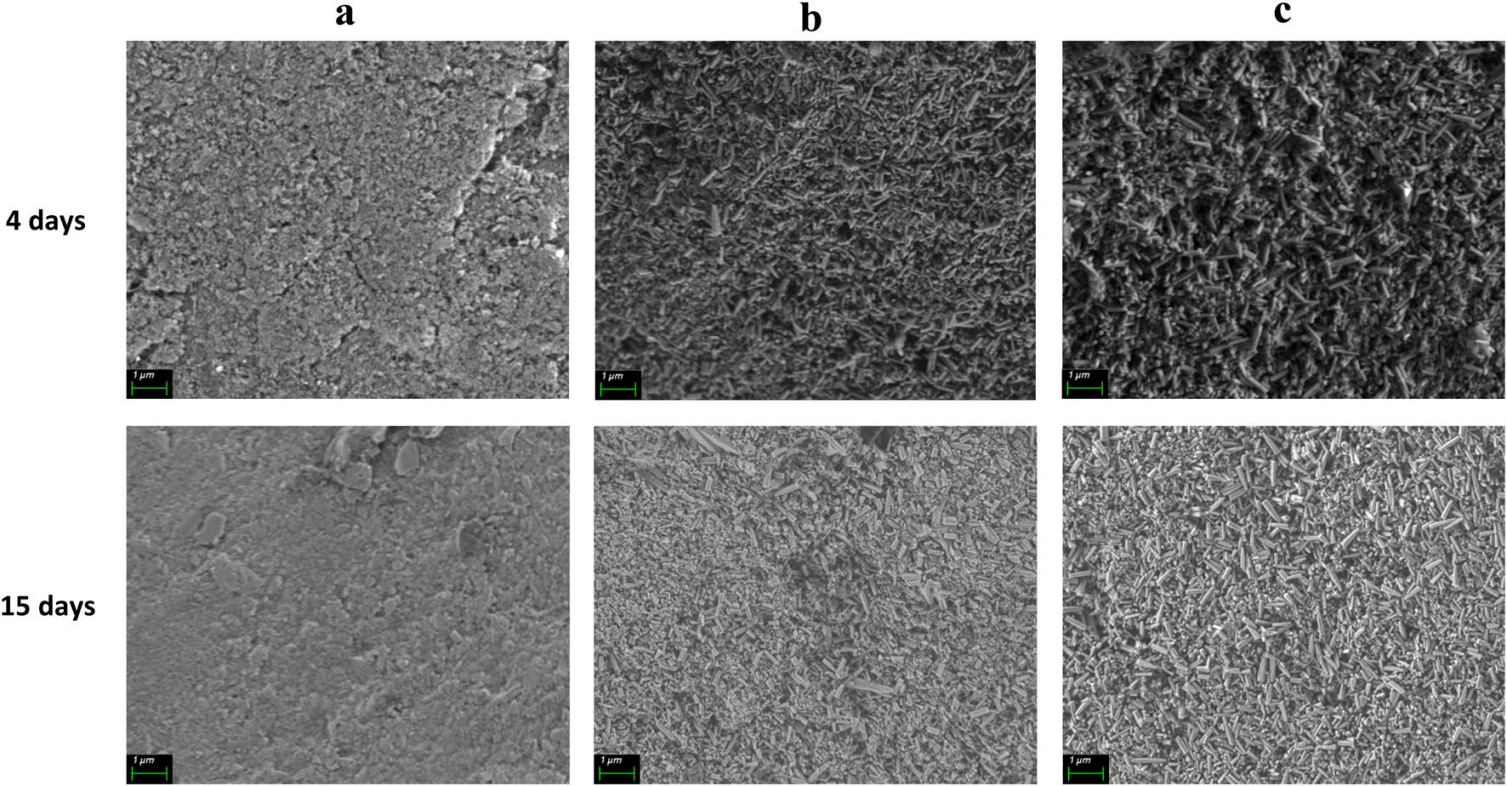
SEM images at × 20,000 magnification level of the tooth surface after 4 and 15 days of treatment: blank (**a**), NMTD (**b**), NMTD and amelogenin (**c**)

The difference between the treatment in the presence and absence of amelogenin was studied. In Fig. 7, it can be appreciated that the crystals are bigger in the presence of amelogenin. The diameter of the crystals for the 4-day treatment is 0.11 ± 0.02 µm for NMTD and 0.14 ± 0.02 µm for NMTD in the presence of amelogenin. In the case of the 15-day treatment, the diameter of the crystals is 0.13 ± 0.02 µm for the treatment without protein and 0.17 ± 0.03 µm with amelogenin. The 15-day treatment with protein shows significant differences in crystal size versus NMTD alone, according to the one-way ANOVA (analysis of variance) test performed with GraphPad Prism 9.

To study the composition of the remineralized layer, an EDX study was performed by measuring blank and treated samples after 4 and 15 days of treatment and comparing them to the references of fluorapatite and hydroxyapatite powder. EDX results are shown in Table 1.

**Table 1:** Surface EDX measurements of the different treatments at different times: blank sample after 4 days (BL4), NMTD-treated sample after 4 days (NMTD4), NMTD+amelogenin-treated sample after 4 days (AH4), blank sample after 15 days (BL15), NMTD+treated sample after 15 days (NMTD15), NMTD-amelogenin-treated sample after 15 days (AH15), fluorapatite powder reference (FA), hydroxyapatite powder reference (HA)

	BL4	NMTD4	AH4	BL15	NMTD15	AH15	FA	HA
Ca (atomic %)	18 ± 5	14 ± 1	20.7 ± 0.1	20 ± 4	14.78 ± 0.01	20.6 ± 0.1	25 ± 4	21 ± 3
P (atomic %)	15 ± 3	10.5 ± 0.5	14.68 ± 0.08	19 ± 2	10.9 ± 0.1	14.53 ± 0.04	14.3 ± 0.8	14.7 ± 0.9
O (atomic %)	66 ± 8	64.8 ± 1.0	56.5 ± 0.4	61 ± 5	63.3 ± 0.5	56.4 ± 0.5	56 ± 2	65 ± 3
F (atomic %)	0.3 ± 0.1	10.8 ± 0.5	8.1 ± 0.2	0.1 ± 0.1	11.0 ± 0.4	8.5 ± 0.4	4.9 ± 0.7	0.00 ± 0.01

**Fig. 8:**
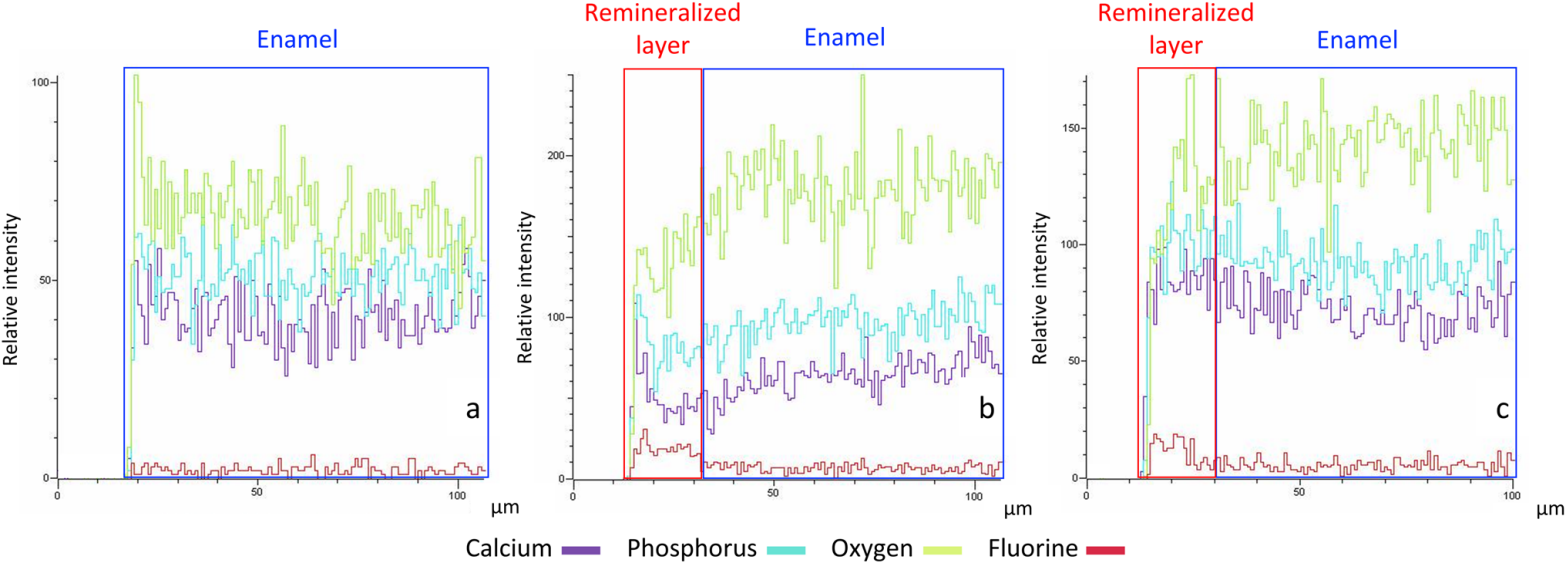
Longitudinal section SEM–EDX analysis of blank sample after 15 days (**a**), NMTD-treated sample after 15 days (**b**), NMTD+amelogenin-treated sample after 15 days (**c**)

To study the evolution of the formed layer across its outer part to the enamel, different EDX measurements were taken in longitudinally cut samples of 15 days through the formed layer until the enamel. Figure 8 shows the variations of each element when moving across the new layer to the natural bovine enamel.

#### Nanoindentation

**Fig. 9:**
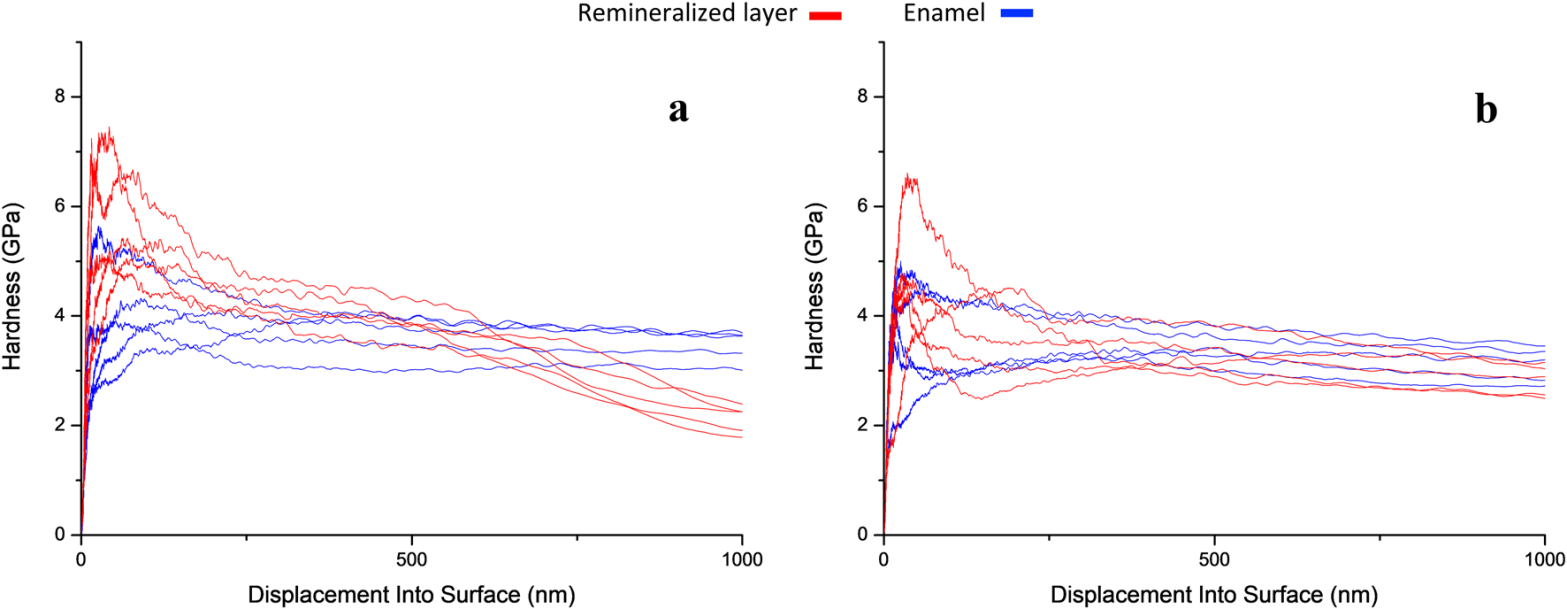
Longitudinal section continuous stiffness measurements of a 15-day NMTD-treated sample (**a**) and a 15-days NMTD+amelogenin-treated sample (**b**)

To assess the hardness of both kinds of remineralized layer with respect to the enamel, CSM measurements were taken in the layer and the enamel of longitudinally cut samples; the measurements are shown in Fig. 9.

## Discussion

The results of the solution experiment confirm the expected formation of fluorapatite. It can be observed in Fig. 4 that the shape of the crystals formed with the NMTD in artificial saliva solution after 24 h resembles the first phase of the formation of fluorapatite spherulites [71–74]. Moreover, the EDX spectrum of the crystals matches the fluorapatite reference spectrum.

The remineralization of the tooth samples has been successful, as it can be seen in Fig. 5, the thickness of the remineralized layer progressively increases until day 15, when it reaches its natural maximum at around 23 ± 1 µm.

Figure 6 shows the remineralized layer of the 15 days treatment to remain intact after brushing for 15 min, demonstrating that it is attached to the tooth surface strongly enough to resist the brushing of the daily hygiene.

The increase in crystal size with amelogenin for both treatment times (4 and 15 days) seen in Fig. 7 suggests amelogenin protein to accelerate the crystallization rate [75].

We can observe in Table 1 that the presence of amelogenin during the remineralization process produces a layer more similar in composition to pure fluorapatite, with more Ca and P but less O and F than the layer formed only with NMTD that also shows the formation of other minerals like calcium fluoride. This calcium fluoride is a precursor that will derive in fluorapatite in the presence of phosphate [2, 76]. The composition of the blank samples treated with artificial saliva alone is similar to that of hydroxyapatite as expected. The results obtained from the 4-day and 15-day treatments are similar, which means that extending the treatment time increases the thickness of the layer, as observed previously, but does not alter its composition.

In Fig. 8, comparing the remineralized layer (first 23 µm) to natural enamel (beneath this 23 µm), fluorine concentration is higher and the oxygen concentration is lower due to the expected production of fluorapatite in the layer (increase of fluoride content) replacing corresponding hydroxyapatite (decrease of the OH^−^ content). In the presence of amelogenin, calcium and phosphorus remain largely similar along the new layer to the original enamel since fluorapatite and hydroxyapatite contain the same amount of calcium and phosphorus, thus supporting our expected mineral formation. On the contrary and in the absence of amelogenin, the observed decrease of oxygen, calcium, and phosphorus in the formed layer against the original enamel may be interpreted by the formation of a considerable proportion of CaF_2_ instead of just fluorapatite. In the blank samples, these changes are not observed due to the absence of the remineralized layer.

We can observe in Fig. 9 the remineralized layer without protein to be slightly harder than enamel during the first 500 nm although it loses hardness from there unlike enamel. However, in the presence of amelogenin, the hardness of the remineralized layer is maintained at values similar to the enamel. These results may be due to the role of amelogenin in guiding the morphology and alignment of crystals formation [22, 43].

The development of technologies to rebuild tooth enamel and preserve tooth structure is of great interest due to the inability of the mature tooth enamel to regenerate itself after substantial mineral loss [1, 2, 25, 40, 47, 77–79]. A widely studied remineralizing system uses nanocomplexes of casein phosphopeptide-amorphous calcium phosphate (CPP-ACP) to stabilize and deliver bioavailable ions [80]. CPP-ACPs have been introduced in toothpastes, mouthwashes, lozenges, chewing gums and even in bovine milk [81–83]. Another option is the combination of CPP-ACP with fluoride or the application of casein phosphopeptide-amorphous calcium fluoride phosphate (CPP-ACFP) to form fluorapatite. CPPs prevent the promotion of dental calculus, which consists of dental plaque mineralization, by stabilizing the calcium, phosphate, and fluoride ions. Yet, the ions are freely bioavailable to induce remineralization by their diffusion through concentration gradients into enamel subsurface lesions [83, 84]. CPP-ACP and CPP-ACFP are the technologies with most supporting evidence to sustain their use among those commercially available for dental remineralization [80]. In the case of the NMTD product, fluorapatite is also formed since these same ions are supplied. The ions are delivered in a controlled manner by means of weak ion-exchange resins, avoiding the precipitation of unwanted compounds by the massive encounter of ions outside the enamel surface, and allowing remineralization with fluorapatite by delivering the ions slowly to the enamel surface. The drawback of this necessary controlled release would be the long application time of the NMTD product required to achieve adequate remineralization, but it avoids high fluoride concentrations in the oral cavity unlike non-controlled-release products.

In conclusion, the NMTD product is effective and induces the remineralization after an acid attack creating a fluorapatite layer of around 23 µm after 15 days of treatment, overcoming the brushing process of the daily hygiene. The presence of amelogenin protein during the remineralization process improves the layer hardness and the crystal morphology, also accelerating the crystallization rate. Furthermore, amelogenin seems to induce the composition of the layer closer to that of pure fluorapatite. The novel product of NMTD with amelogenin induces complete remineralization with a hardness that reaches the levels observed in natural healthy enamel. Therefore, the novel product is promising to provide long-term remineralization to inhibit caries and protect tooth structures. Nevertheless, further studies would be necessary to evaluate the clinical applicability of this biomimetic material.
